# Towards real-time PGS range monitoring in proton therapy of prostate cancer

**DOI:** 10.1038/s41598-021-93612-y

**Published:** 2021-07-28

**Authors:** Paulo Magalhaes Martins, Hugo Freitas, Thomas Tessonnier, Benjamin Ackermann, Stephan Brons, Joao Seco

**Affiliations:** 1 German Cancer Research Center - DKFZ, Heidelberg, Germany; 2 Instituto de Biofísica e Engenharia Biomédica, Faculdade de Ciências da Universidade de Lisboa, Lisbon, Portugal; 3 Departamento de Física e Astronomia, Faculdade de Ciências da Universidade do Porto, Porto, Portugal; 4 Heidelberg Ion-Beam Therapy Center (HIT), Department of Radiation Oncology, Heidelberg University Hospital, Heidelberg, Germany; 5 Department of Physics and Astronomy, University of Heidelberg, Heidelberg, Germany

**Keywords:** Prostate, Prostate cancer, Applied physics, Techniques and instrumentation, Biomedical engineering, Radiotherapy

## Abstract

Proton therapy of prostate cancer (PCPT) was linked with increased levels of gastrointestinal toxicity in its early use compared to intensity-modulated radiation therapy (IMRT). The higher radiation dose to the rectum by proton beams is mainly due to anatomical variations. Here, we demonstrate an approach to monitor rectal radiation exposure in PCPT based on prompt gamma spectroscopy (PGS). Endorectal balloons (ERBs) are used to stabilize prostate movement during radiotherapy. These ERBs are usually filled with water. However, other water solutions containing elements with higher atomic numbers, such as silicon, may enable the use of PGS to monitor the radiation exposure of the rectum. Protons hitting silicon atoms emit prompt gamma rays with a specific energy of 1.78 MeV, which can be used to monitor whether the ERB is being hit. In a binary approach, we search the silicon energy peaks for every irradiated prostate region. We demonstrate this technique for both single-spot irradiation and real treatment plans. Real-time feedback based on the ERB being hit column-wise is feasible and would allow clinicians to decide whether to adapt or continue treatment. This technique may be extended to other cancer types and organs at risk, such as the oesophagus.

## Introduction

Range verification is one of the most important problems to be solved in particle therapy^1,2^. Offline positron emission tomography (offline PET) has verified range uncertainties of approximately 6 mm^3^. Offline PET scans are performed after irradiation, and the activated tissue is imaged. However, this technique suffers from low signal and biological washout over time. More recent results with in-beam PET have demonstrated the online capabilities of this technique^4^. Prompt gamma imaging (PGI) has emerged as an alternative that relies on the prompt nature of the gamma radiation emitted during particle therapy. Range verification can be accomplished in real time during treatment, thus providing a means to avoid unwanted irradiation of healthy tissues. Since 2006, several concepts based on imaging and non-imaging systems have been developed^5–11^. Eventually, two of them—the knife-edge slit camera and prompt gamma spectroscopy—reached the clinical phase^12,13^ and are currently being used at proton facilities.

Proton therapy for prostate cancer (PCPT) has been a reality since the 1990s^14,15^. Several clinical studies have estimated the toxicity of prostate cancer therapy with photons^16,17^, protons^14,18–23^, and carbon ions^24,25^. At the outset, PCPT was considered to deliver less dose than photon radiation to normal tissues surrounding the prostate, such as the rectum and bladder^26–28^. PCPT had, however, a major setback, with two clinical studies reporting higher toxicity than conventional photon therapy^19,21^. Sheets *et al.* showed that although intensity-modulated radiation therapy (IMRT) delivered three times more radiation to the body, it presented 50% less gastrointestinal morbidity. Proton therapy-treated patients were more likely to receive a diagnosis of gastrointestinal morbidity and undergo gastrointestinal procedures. There were, however, no significant differences in urinary nonincontinence or incontinence diagnoses or procedures, erectile dysfunction, or hip fractures^21^. Kim *et al.* also showed that proton therapy had the highest rate of grade 3/4 toxicity among radiotherapy modalities (20.1 per 1000 person-years)^19^. However, the authors pointed out that the sample size for the proton cohort was quite small because the study included patients diagnosed from 1992 to 2005, a period when proton therapy was in its relative infancy and only passively scattered proton therapy (PSPT) was available. In the meantime, intensity-modulated proton therapy (IMPT) was developed both for protons^29,30^ and carbon ions^31^. More recent studies have demonstrated more favourable toxicity outcomes with proton therapy^20,22,23^.

Another drawback of PCPT is the irradiation of the femoral heads, which lay in the path of the beam to the prostate. These structures influence the stopping power due to their high density. This effect is even more pronounced in the presence of prosthetic hips, where proton therapy is not recommended at all. Anterior-oblique (AO) fields have been proposed to circumvent this problem with proton^32,33^ and carbon beams^34^. However, the target coverage is substantially reduced after considering interfractional variations in AO plans, thus increasing the susceptibility to underdosing^33^. Adaptive range verification could play a decisive role in recovering the target coverage. In these cases, superior sparing of the rectum was demonstrated. For this purpose, an in vivo diode-based range verification system was developed and commissioned at the Massachusetts General Hospital (MGH)^35,36^. This system was designed for passive-scattering proton delivery and relies on a $3 \times 4$ matrix of 1 mm diodes mounted in a water balloon. An accuracy on the order of 1 mm for the water equivalent path length (WEPL) measurements was demonstrated. The rectal wall was shown to receive doses of 1.6% for anterior fields and 0.4% for AO fields^36^. Another technique to improve gastrointestinal toxicity and mitigate the uncertainties in proton relative biological effectiveness (RBE) is the use of rectal hydrogel spacers located between the prostate and the rectum^37–39^. This is, however, an invasive technique that demands surgery, as the spacers are later absorbed.

Endorectal balloons (ERBs) have long been used to stabilize the prostate location^40,41^, especially during radiotherapy with photons, such as three-dimensional conformal radiotherapy (3D CRT)^40,42,43^ and intensity-modulated radiotherapy (IMRT)^44–47^. Clinical studies using ERBs in IMRT evaluated acute toxicity^48,49^ and rectal wall sparing^50^. Reduction in the dose to the rectal wall by means of an ERB has been observed by several authors^40,51,52^.

Among the range verification techniques, prompt gamma spectroscopy (PGS) has demonstrated the ability to measure absolute range deviations during the course of treatment. This technique relies on the analysis of the prompt gamma energy spectrum, which is characterized by specific energy lines corresponding to the reaction channels of the irradiated protons with the elements of the human body. The most common reactions are those with oxygen and carbon atoms, which become excited and eventually emit prompt gamma rays up to 10 MeV^6,53^. However, other reactions emitting low-energy prompt gammas following the irradiation of metals by protons^54^ and helium ions^55^ have been shown.

We describe the implementation of an ERB inflated with a mixture of water and silicon dioxide (SiO$_2$) to wirelessly monitor the proton range in PCPT via prompt gamma rays. The presence of silicon in the mixture allows the differentiation of the characteristic gamma emission lines resulting from the bombardment of the $^{28}$Si atoms by protons. The cross-sections associated with such reactions have been investigated^56–59^. A gamma line emitted at 1.78 MeV provides a unique signature for differentiation. Instantaneous feedback from the ERB being hit by protons allows for prompt binary output regarding the irradiation of the rectum.

We irradiated single spots with beams, hitting only water or other water mixtures and solutions inside the ERB. We evaluated a worst-case scenario of an ERB surrounded by a phantom filled with water that emits prompt gamma rays under irradiation, with energy lines strongly competing with those of interest in the ERB. The water flasks in the beam path played a stronger role in signal deterioration. The prompt gamma attenuation within the water flasks placed in the path from the interaction point to the detectors was less pronounced. Finally, we evaluated two real treatment plans with a single 2 Gy anterior field and a 1 Gy AO field. A realistic phantom with an inserted ERB filled with the SiO$_2$ water mixture was irradiated with active pencil beams. A scenario with the plan overlapping the ERB and another one with the real plan were considered. The iso-energy layers (IELs) crossing the balloon were identified, as well as the columns parallel to the ERB within each IEL.

## Results

**Figure 1:**
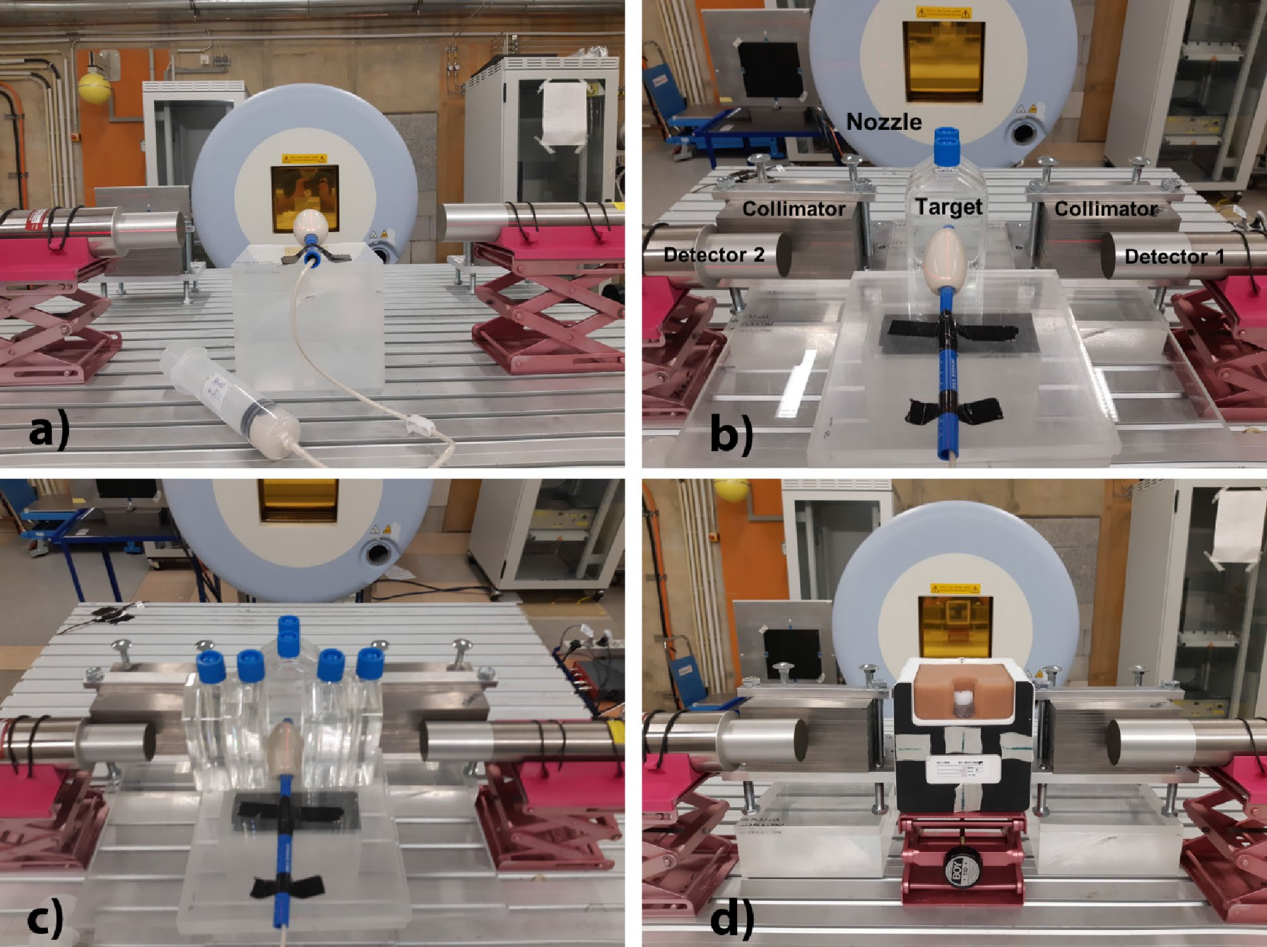
Experimental setup. (**a**) Photo of the experimental setup, consisting of an irradiating nozzle, a target, and two CeBr$_3$ detectors. (**b**) Photo of a similar setup with the target placed behind two flasks of water in the beam path. (**c**) That with two flasks of water placed on each side of the target. Two tungsten collimators were placed in front of each detector in the beam direction. (**d**) Photo of the same setup with the target of a prostate phantom with a commercial silicone insert.

We started by irradiating different water solutions and mixtures with single-spot proton beams. Figure 1 shows the detectors, the targets, and the beam nozzle. Figure 1a shows an ERB filled with a water mixture to be irradiated with the lowest energy available (48 MeV). Afterwards, we increased the energy of our proton beam to an energy applicable in PCPT. Figure 1b shows two flasks of water in front of our target. To evaluate the prompt gamma attenuation in the patient, we placed two water flasks on each side of the target, i.e., in the path from the target to the detectors, as shown in Fig. 1c. Figure 1d shows a prostate phantom with a custom-made insert filled with a commercial silicone sealant. Two tungsten collimators were placed in a semi-collimation configuration in front of each detector in the beam direction to prevent scattered particles in the nozzle from hitting the detectors and to collimate the prompt gammas only from the most downstream region. These collimators had a strong impact in reducing the detector count rate, thus allowing higher beam intensities.

**Figure 2:**
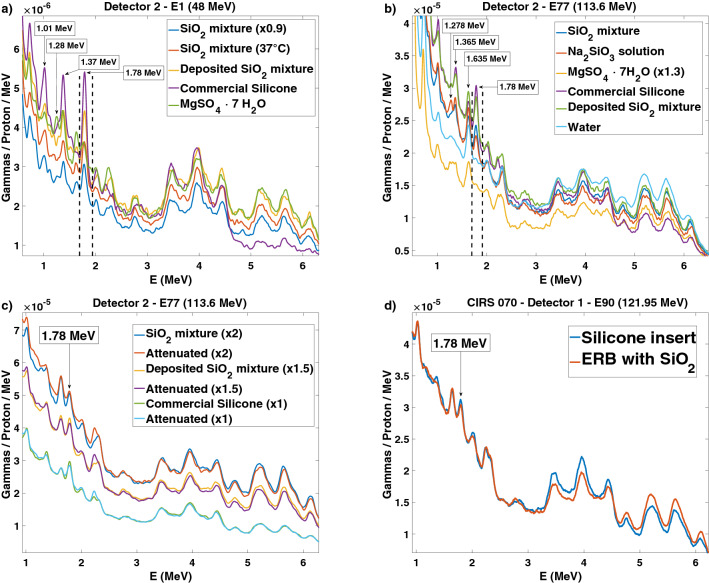
Energy spectra obtained from the irradiation of several targets by protons. (**a**) The targets irradiated with the minimum proton energy exhibit very prominent silicon energy peaks. A sulphur energy peak at 1.28 MeV is also visible. (**b**) Targets located after two flasks of water were irradiated with an energy of 113.6 MeV. The silicon energy peak at 1.78 MeV exhibits relatively low prominence for all materials and is absent for water. A sodium energy peak at 1.278 MeV is also visible. (**c**) The effect of the prompt gamma attenuation by the two flasks of water on each side of the target in the path to the detectors is shown for the three targets. (**d**) The commercial silicone insert and the balloon with the mixture of SiO$_{2}$ inserted in the prostate phantom present similar spectra under irradiation by proton beams of 121.95 MeV.

In Fig. 2a, we show the energy spectra of several water solutions and mixtures irradiated by single-spot proton beams at the lowest energy. The mixture with silicon dioxide (SiO$_2$) exhibits several differences from the other solutions. The solution of heptahydrate magnesium sulphate (MgSO$_4\cdot$7H$_2$O), also known as Epsom salt, responds to higher temperatures with higher solubility. This is not observed in the SiO$_2$ mixture. The addition of sodium hydroxide (NaOH) to the SiO$_2$ mixture creates a solution of sodium metasilicate (Na$_2$SiO$_3$), but the quantity in grams of dissolved solute remains the same as that in the mixture. The limit for SiO$_2$, either mixed or dissolved in 60 mL of water, is 40 g. Above that quantity, the viscosity increases, and the mixture or solution cannot flow inside the small diameter tube between the syringe and the balloon. A commercial silicone sealant was also irradiated for the sake of comparison with the expected silicon gamma lines. Figure 2b shows the spectra of these targets with two water flasks placed in front of them. Due to the increased lateral spread and range straggling, the 1.78 MeV silicon gamma line is smeared out, and the nearby 1.635 MeV energy line resulting from oxygen irradiation becomes more prominent. The addition of NaOH creates a sodium line at 1.278 MeV, increases the oxygen and sodium lines at 1.635 MeV, and decreases the silicon line at 1.78 MeV. In view of these results and due to the simplicity of operation and its harmlessness (lack of toxic effects), we decided to continue our studies with a mixture of water and SiO$_2$. Figure 2c shows the spectra obtained with two water flasks placed on each side of the target. The prompt gamma attenuation effect is hardly visible. All sequential effects were combined, thus mimicking a worst-case scenario of a target mostly made of water. In this case, the prompt gamma water lines compete strongly with the prompt gamma silicon lines. Figure 2d compares the energy spectra from the proton irradiation of a prostate phantom with either a silicone insert or an ERB filled with a water mixture of SiO$_2$. The differences in the prominences of the peaks of interest are negligible.

**Figure 3:**
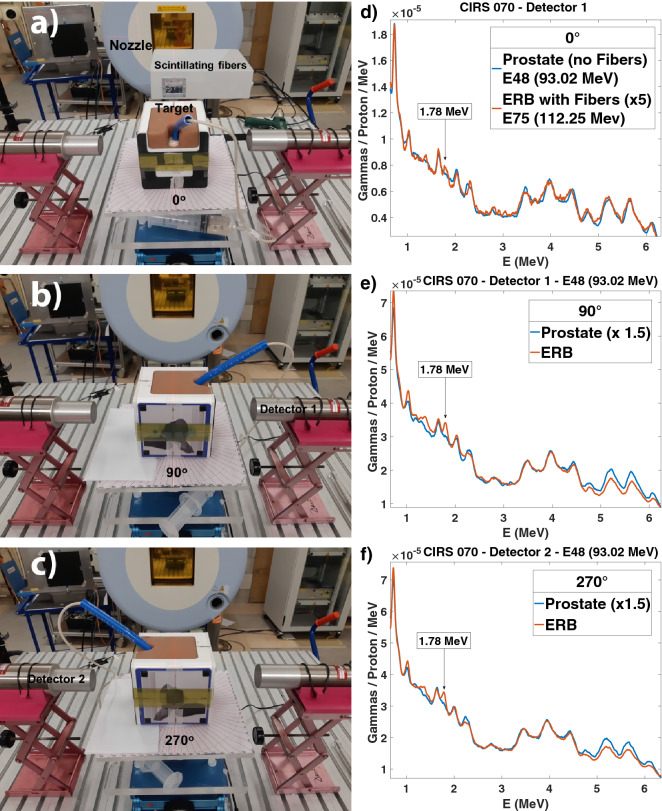
Prostate phantom irradiated at different gantry angles. (**a**–**c**) Photos of the prostate phantom with an ERB filled with SiO$_{2}$ and lying on a rotating platform to reproduce the three gantry angles of 0$^\circ$, 90$^\circ$, and 270$^\circ$. (**d–f**), Energy spectra from the irradiation of the prostate and the rectal balloon at the three angles. The silicon energy peak of 1.78 MeV is clearly distinguishable in the ERB spectra.

We then aimed to evaluate the cumulative effects of range straggling and prompt gamma attenuation in a prostate phantom with an inserted ERB filled with a mixture of water and SiO$_2$. Therefore, we irradiated the prostate and the ERB with single-spot proton beams at different phantom positions. To reproduce a real treatment scenario within a rotating gantry, the phantom was rotated by 90$^\circ$ in the transaxial direction and irradiated by a horizontal beam. Figure 3a–c shows the phantom at three gantry angles: 0$^\circ$, 90$^\circ$, and 270$^\circ$. Figure 3d–f shows the spectra resulting from the irradiation of the prostate and the ERB with single spot beams in the three positions. A 1.78 MeV silicon line is present in the ERB irradiation and absent in the prostate irradiation. For the lateral beams, the closer the ERB is to the detector, the better the signal from the 1.78 MeV prompt gammas. Detector 1 collects a higher signal for the 90$^\circ$ angle, while detector 2 collects a higher signal for the 270$^\circ$ angle. To increase the signal at a 0$^\circ$ angle, we used the timing information of the arrival time of the protons provided by the scintillating fibres placed between the nozzle and the target^60^. The trigger was not further used in the treatment plans due to the strong impact in the statistics and due to the intensity constraints (increasing pile-up above $8\times 10^7$ p/s).

**Figure 4:**
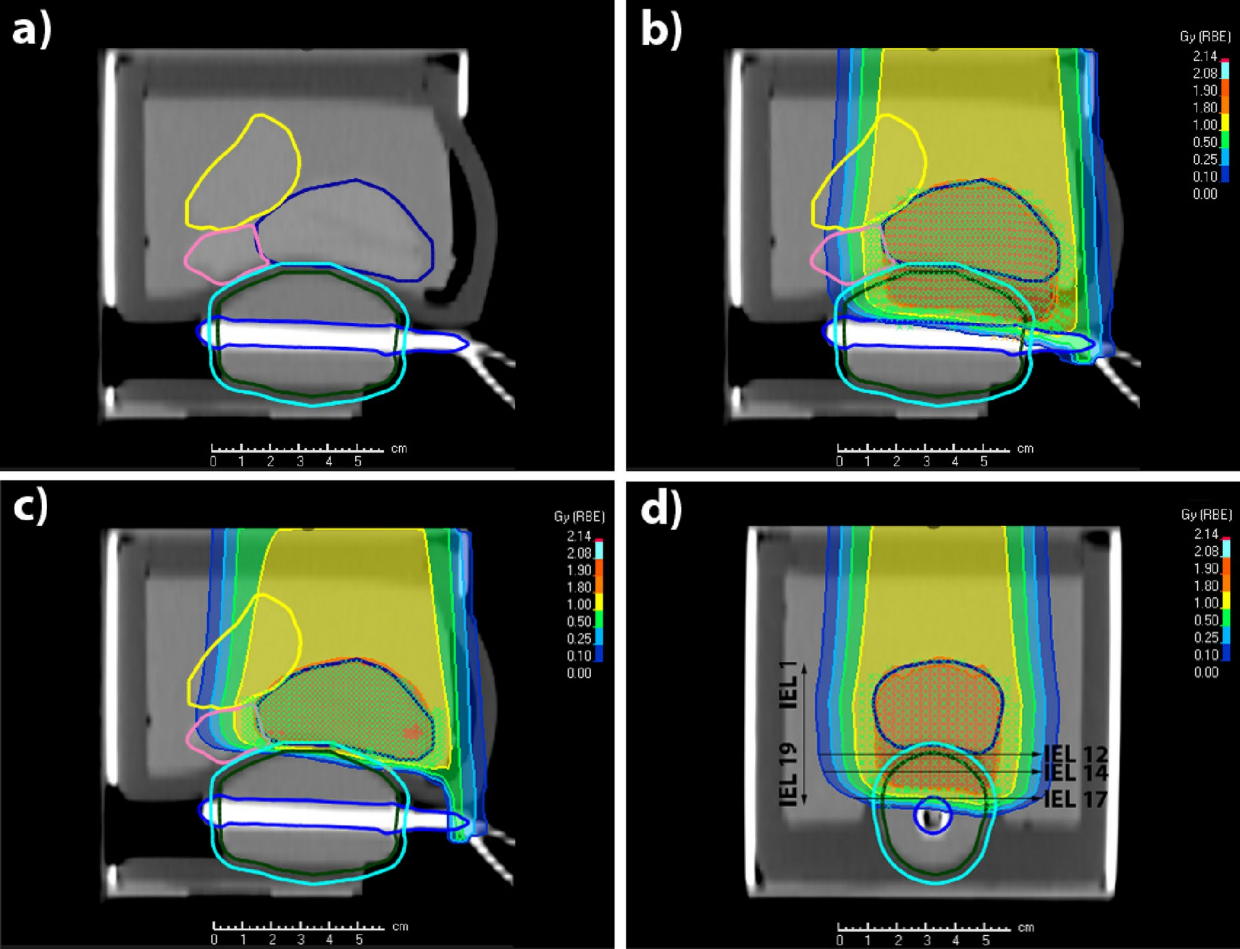
Phantom computed tomography and anterior proton plans. (**a**) Sagittal view of a computed tomography of the prostate phantom with the ERB. The spacing between the prostate and the ERB is visible as well as the seminal vesicles and the bladder. (**b–c**) Sagittal view of two anterior plans either overlapping with the ERB or conforming to the prostate. (**d**) Coronal view of the overlapping plan where all 19 IELs are present. All spots in the distal IELs overlap with the ERB, while the proximal IELs (12–14) just overlap with its central part.

In the last setup, we considered real treatment-like plans. Figure 4 shows the computed tomography (CT) and the plans of an anterior beam irradiating the prostate either conformally or overlapping with the ERB. In Fig. 4a–c, the sagittal views through the prostate and the ERB clearly show their structure and the spacing between the ERB and the prostate. The CT also shows the seminal vesicles, the bladder, and the small tube inside a larger tube that transports the solution or mixture from the syringe to the ERB. Figure 4d shows a coronal plan where the IELs as well as the spots overlapping the prostate and the ERB are visible. While IEL 17 has all spots overlapping within the ERB, IEL 12 only has six central overlapping spots.

**Figure 5:**
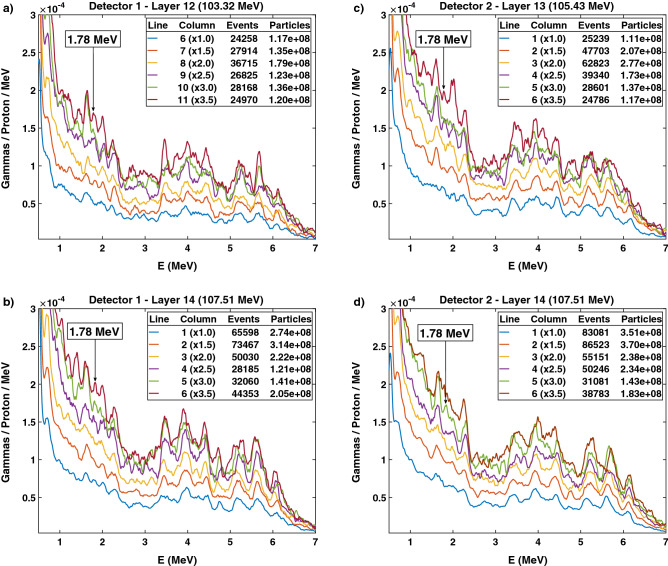
Energy spectra from the irradiation of the prostate phantom according to an anterior proton plan overlapping with the ERB. (**a,b**) Detector 1 detects the prompt gammas emitted from the irradiation of IELs 12 and 14. (**c,d**) Detector 2 detects the prompt gamma emitted from the irradiation of IELs 13 and 14. The plots are reordered into columns such that the central columns present a silicon energy line of 1.78 MeV. Detectors 1 and 2 detect the columns to the left and the right in the BEV, respectively, with higher sensitivity. For both detectors, the central columns, despite the lower counts due to attenuation, present silicon energy lines of 1.78 MeV.

Our goal was to determine at which IEL the protons hit the ERB with the overlapping anterior beam. However, since not every spot within each IEL overlapped with the ERB, we sorted the irradiation within each IEL by columns parallel to the ERB and attributed time stamps to each column. Figure 5 shows the prompt gamma spectra from the irradiation of the phantom at IELs 12, 13, and 14. IEL 12 is at the interface between the prostate and the ERB. Columns were detected from the first to the last starting in beam-eye view (BEV) on the left for detector 1 and on the right for detector 2. While detector 1 detects the columns to the left in BEV with higher sensitivity, detector 2 has a higher count rate for columns to the right in BEV. For detector 1, we observe a 1.78 MeV silicon line emerging from column 8 to column 11 at IEL 12 (103.22 MeV). At IEL 14 (107.51 MeV), a 1.78 MeV silicon energy line emerges from column 4. For detector 2, this energy line emerges from column 4 for IEL 13 (105.42 MeV) and column 3 for IEL 14. The number of protons per column is on the order of 1–3.7 $\times$ 10$^8$, and the detected prompt gammas per column are on the order of 25-85 kcts.

**Figure 6:**
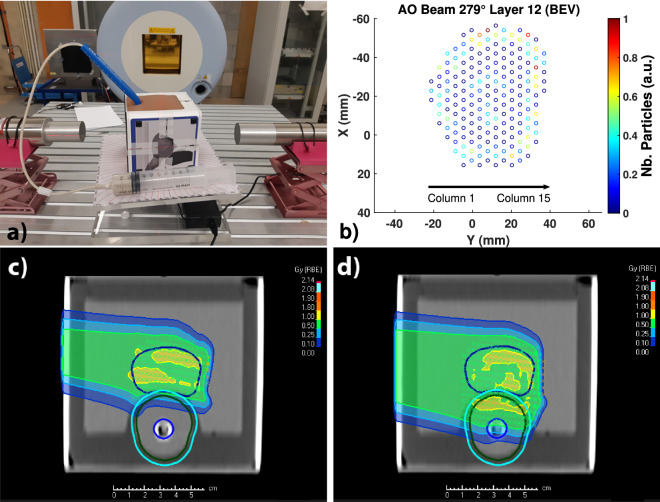
Anterior-oblique plan for a 279$^\circ$ gantry angle. (**a**) Photo of the prostate phantom lying on a rotating platform commanded remotely from the control room. The ERB was closer to detector 2. (**b**) Schematics of the irradiation of each IEL in a column-wise fashion. The *Y*-coordinates represent the horizontal direction and increase from left to right in the BEV, and the *X*-coordinates represent the vertical direction and increase from top to bottom. (**c**) Plans of the AO beam conforming to the prostate (**d**), and that overlapping with the ERB.

In the AO plan, we also reordered each IEL of the plan in such way that they were irradiated in columns parallel to the ERB from left to right in the BEV. Figure 6a and b shows a photo of the prostate phantom at an angle of 279$^\circ$ and schematics of the irradiation of IEL 12 from column 1 to column 15. The plans with and without overlap with the ERB are shown in Fig. 6c and d.

**Figure 7:**
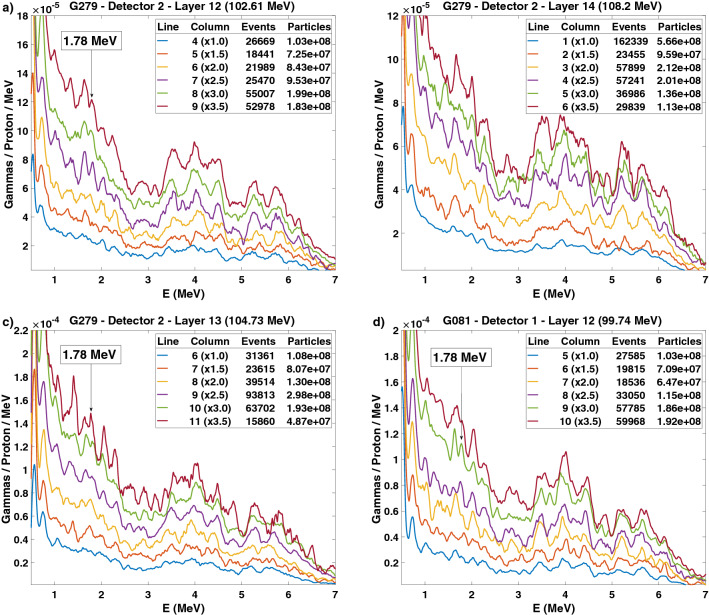
Energy spectra from the irradiation of the prostate phantom by an AO plan. (**a**) Detector 2 detects a 1.78 MeV silicon line starting at column 6 of the IEL 12 (102.6 MeV) during irradiation with 8.4 $\times$ 10$^7$ protons, thus indicating the entrance of protons into the ERB. (**b**) The real plan without overlap with the ERB presents no silicon energy peaks for the last columns of IEL 14 (108.2 MeV). (**c**) Another measurement separated in time by one month also shows a 1.78 MeV silicon peak for the AO beam overlapping with the ERB. (**d**) A symmetric configuration with an AO plan for an 81$^{\circ }$ gantry angle results in the detection of prompt gamma radiation by the first detector with the same energy lines of interest.

In Fig. 7a, we observe that the columns to the right overlapping with the ERB produce a 1.78 MeV prompt gamma line, while those to the left irradiate the prostate and therefore present no such line. Such tracking is possible with columns comprising less than 10^8^ protons. For IEL 12, the protons start hitting the ERB at column 6 with $8.4 \times 10^7$ particles. In Fig. 7b, we confirm that the real plan without overlap with the ERB does not yield a 1.78 MeV energy line for the last columns to the right. For the sake of irradiation speed, the first depicted column aggregates several columns to the left in the prostate region. An independent measurement undertaken after one month with the same gantry angle demonstrates the existence of 1.78 MeV energy lines for the columns overlapping with the ERB (Fig. 7c). An additional measurement at a symmetric position of 81$^\circ$ shows 1.78 MeV energy lines for the columns to the left closer to detector 1 (Fig. 7d).

**Figure 8:**
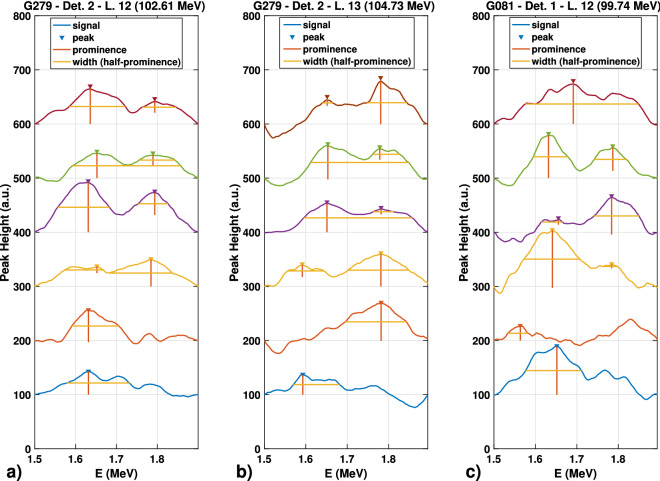
Analysis of the region of interest for the energy spectra obtained during irradiation of the prostate phantom by the AO plans. (**a**) The 1.635 MeV and 1.78 MeV peaks are more prominent in the four top spectra. These peaks correspond to columns 6 to 9 of IEL 12 (Fig. 7a). (**b**) The four top spectra presenting the peaks of interest correspond to columns 8 to 11 of IEL 13 (Fig. 7c). (**c**) The gantry angle of 81$^{\circ }$ also presents the peaks of interest for columns 7 to 9 of IEL 12 (Fig. 7d). The vertical scale has been adapted for visualization purposes.

A peak analysis within the region of interest for the spectra presented in Fig. 7 is depicted in Fig. 8. The prominence and the width at half prominence are shown for the peaks of interest. The top four peaks that result from the irradiation of the ERB are indicative of the prompt gamma lines associated with the reaction between the protons and the silicon atoms.

## Discussion

Prompt gamma spectroscopy (PGS) is currently one of the most promising techniques for particle range monitoring and measurements of the elemental composition of irradiated targets in particle therapy^6,13,55,61^. This technique facilitates absolute range measurements with millimetre precision due to accurate knowledge of the nuclear reaction cross-sections between the irradiated particles and the types of atoms in the patient. Two PGI modalities, PGS and the knife-edge slit camera, have now reached the level of clinical prototypes^12,13^. The combination of in vivo range monitoring and adaptation methods has been proposed for the treatment of prostate cancer with either anterior beams^35^ or anterior oblique (AO) beams^33^. An in vivo range verification system has already been commissioned^36^. This system is composed of a 4 by 3 array matrix of silicon diodes attached by a self-adhesive surface to an ERB and presents a WEPL measurement accuracy on the order of 1 mm.

In this paper, we propose a wireless solution that uses prompt gamma rays to monitor the interaction of protons within an ERB filled with a silicon dioxide water mixture and inserted in a prostate phantom. This concept aims to monitor the proton range in PCPT in real time. The irradiation of atomic nuclei within the human body by protons emits prompt gamma rays with characteristic energy lines^6,56^. The irradiation of carbon and oxygen atoms is followed by the emission of prompt gamma radiation with low and high energies (0.511 MeV, 0.718 MeV, 1.022 MeV, 1.635 MeV, 2.31 MeV, 2.8 MeV, 4.4 MeV, 5.2 MeV, and 6.1 MeV)^6,54^. Conversely, during the irradiation of metals, prompt gamma rays are emitted with lower energy (below 3 MeV)^54,55^. This radiation exits the patient under proton bombardment and may be detected by scintillating crystals, e.g., CeBr$_3$. The signals are digitally converted and processed to extract energy and time information.

Metals usually not present within the human body are good candidates for ranging probes. Although not a metal, silicon dioxide has been shown to be a good choice due to the unique signature provided by the emission of a prompt gamma energy line at 1.78 MeV. This line is distinguishable from the remaining spectrum and can therefore provide binary information about the elemental composition of the material being hit. However, even with good dose confinement to the target, the patient is still exposed to a dose in the organ at risk (OAR) and very likely prompt gammas emitted from the ERB. Therefore, a possible solution would be to set a threshold on the 1.78 MeV prompt gammas detected at a certain IEL and neighbouring IELs. This binary output might trigger a decision on whether to continue or stop/adapt the treatment since an organ at risk may be endangered.

Proton beam delivery with spot- or raster-pencil-beam scanning (PBS) is particularly suitable for such an approach. A synchronization between beam delivery and prompt gamma detection may allow real-time monitoring of the voxels being hit and simultaneous comparison to the prediction. A standard 2 Gy prostate treatment provides sufficient statistics for such monitoring. Due to the round shape of the rectum, an anterior beam requires column-wise delivery parallel to the rectum so that which IEL column the nuclear reactions with the silicon take place in can be inferred. The range monitoring also requires detectors closer to the irradiated column. Therefore, the right columns in the beam-eye view require detectors on the right side, and the left columns are better detected by detectors on the left side. The AO beams present an even more preferable solution, as the geometry allows the detectors to be placed closer to the ranging probe. All columns within IELs overlapping with the range probe are prone to be detected with higher sensitivity. In the case of a range probe located in the rectum or the oesophagus, the AO beams are especially suitable, as the detector may be positioned at right angles with the patient and close to the probe.

Range monitoring by means of PGS is feasible in PCPT. Once the proton range is under control, one may use fields other than the commonly used bilateral opposing fields that are more robust to range uncertainties. The two AO beams may assume variable angles due to the flexibility provided by the method presented in this paper. Therefore, SiO$_2$-filled ERB combined with PBS delivery and PGS monitoring may allow AO beams to be sensitive against rectal changes within and between treatment fractions.

## Methods

### Prostate phantom

The phantom was a prostate training phantom, CIRS model 070L (CIRS Inc., Norfolk, USA). This phantom is commonly used for ultrasound images and biopsy through the ZSkin rectal wall or perineal membrane. The main inner composition is Zerdine. It includes a urethra with a diameter of 0.7 cm, seminal vesicles with a diameter of 0.7 cm and length of 10 cm, and two lesions. The container has a volume of 9 cm $\times$ 10 cm $\times$ 10 cm and a probe opening of 1.2 cm.

### Endorectal balloon

The endorectal balloon (ERB) was a QLRAD Rectal Pro75 (QLRAD International, Larnaca, Cyprus), which is commonly used to stabilize prostate movement in radiotherapy. It is coupled to a syringe via a smaller tube, and a latch closes the liquid flow. Each ERB was filled with 50 mL. Two ERBs operated over six shifts of six hours with different water solutions.

### Water solutions and mixtures

The mixture of water and silicon dioxide (SiO$_2$) consisted of 90 mL of deionized water and 60 g of diatomaceous earth (*Kieselgur*) from Health Leeds (Health Leeds UK Ltd, Horeb, UK). The solution of sodium metasilicate (Na$_2$SiO$_3$) consisted of 60 mL of water, 27 g of sodium hydroxide, and 40 g of diatomaceous earth. The magnesium sulphate solution consisted of 90 mL of water and 100 g of MgSO$_4\cdot$7H$_2$O (*Epsom salts*) from Health Leeds previously heated to 40 $^{\circ }$C.

### The HIT facility

The Heidelberg Ion-Beam Therapy Center (HIT)^62^ accelerates proton, helium, carbon, and oxygen ions from 48 MeV/u to 430 MeV/u. While protons and carbon ions are routinely implemented in the clinical setting, helium ions are currently being commissioned^63,64^, and oxygen ions remain a research beam species.

The intensities in clinical practice range from 2 $\times$ 10$^6$ p/s for carbon ions to 3.2 $\times$ 10$^{9}$ p/s for protons. There are two horizontal beam rooms and a 360$^{\circ }$ gantry for therapy. There is a horizontal experimental room where all the experiments referred to in this paper were performed.

### Computed tomography

The computed tomography (CT) followed the routine CT protocol for ion beam therapy planning at HIT with the Siemens SOMATOM Confidence RT Pro (Siemens Healthineers, Erlangen, Germany). The phantom and the inserted ERB were scanned with a tube voltage of 120 kV, and the image was reconstructed for a field of view (FOV) of 50 cm with a convolution kernel B40s and a spacing between slices of 3 mm.

### Plans

The treatment-like plans were optimized using a RayStation 10A (RaySearch Laboratories, Stockholm, Sweden) and calculated with a Monte Carlo algorithm. The plans conforming to the prostate (either with 2 oblique fields mimicking AO 279 and 81 degree beams or a single AP beam at 0 degrees) were designed to prevent any proton Bragg peak localization within the ERB and for a maximum rectum dose below 0.3 Gy (RBE) per fraction. The plans overlapping with the ERB for the same beam configurations covered an extended target including the prostate and 1.5 cm extension in the posterior direction (towards the ERB). The dose prescriptions to the targets were a median dose of 2 Gy (RBE) per fraction.

### Experimental setup

The main components of the experimental setup were the nozzle, the target, and the CeBr$_3$ detectors. These detectors are scintillation detectors with very good time and energy resolution. They feature a measured energy resolution of 3.49%^55^ and a measured time resolution of 0.85 ns^60^. They are mainly used for range verification of the proton and ion beams in a patient. The CeBr$_3$ detectors were aligned with the isocentre and positioned at a distance of 15 cm from the beam axis. The CeBr$_3$ crystals are identical in size (diameter d = 3.81 cm and length l = 7.62 cm). One crystal was coupled to a Hamamatsu R13089 photomultiplier tube (PMT), and the other crystal was coupled to a Hamamatsu R9420-100 PMT. Both detectors were plugged to a voltage divider. The anode output fed the data acquisition system (DAQ)^65^. This is a module of a FlashCam FADC system, originally designed for the Cherenkov Telescope Array (CTA)^66^.

### Intensities, acquisition times, and counts

The results shown in Figs. 2 and  3 were obtained with an intensity of 2 $\times$ 10$^8$ p/s and the spill time lasted 1:07 min (14 spills). A total of 1.36 $\times$ 10$^{10}$ protons were delivered and the counts were variable. The results shown in Fig. 5 were obtained with an intensity of 2 $\times$ 10$^8$ p/s and the spill time lasted 2:59 min. A total of 3.43 $\times$ 10$^{10}$ protons were delivered and a total of 7.8 Mcts were recorded. The results shown in Fig. 7a were obtained with an intensity of 2 $\times$ 10$^8$ p/s and the spill time lasted 1:33 min. A total of 1.79 $\times$ 10$^{10}$ protons were delivered and a total of 5.1 Mcts were recorded. The results shown in Fig. 7b were obtained with an intensity of 2 $\times$ 10$^8$ p/s and the spill time lasted 1:11 min. A total of 1.36 $\times$ 10$^{10}$ protons were delivered and a total of 3.8 Mcts were recorded. The results shown in Fig. 7d were obtained with an intensity of 2 $\times$ 10$^8$ p/s and the spill time lasted 1:35 min. A total of 1.83 $\times$ 10$^{10}$ protons were delivered and a total of 5.7 Mcts were recorded.

### Peak analysis

The presence or absence of the silicon line could not be visually verified. Therefore, a simple method was developed to identify the presence of 1.635 MeV and 1.78 MeV peaks within a region of interest. We subtracted the background from the peaks by fitting a straight line through their high and low energy values. The MATLAB function *findpeaks* was adapted to identify the peaks within a certain energy interval and to meet certain criteria. The parameters, such as the minimum peak height or prominence, the minimum peak width at half prominence, and the maximum and minimum distances between energy peaks, were adjusted after the spectra were properly calibrated. Other methods, such as that presented by Dal Bello *et al.*^55^, could also have been used.
